# Evaluation of new farming technologies in Ethiopia using the Integrated Decision Support System (IDSS)

**DOI:** 10.1016/j.agwat.2016.07.023

**Published:** 2017-01-31

**Authors:** Neville Clarke, Jean-Claude Bizimana, Yihun Dile, Abeyou Worqlul, Javier Osorio, Brian Herbst, James W. Richardson, Raghavan Srinivasan, Thomas J. Gerik, Jimmy Williams, Charles A. Jones, Jaehak Jeong

**Affiliations:** a The Norman Borlaug Institute for International Agriculture, Texas A&M AgriLife Research, College Station, TX, United States; b Department of Agricultural Economics, Texas A&M University, College Station, TX, United States; c Spatial Sciences Laboratory, Texas A&M University, College Station, TX, United States; d Blackland Research Center, Texas A&M AgriLife Research, Temple, TX, United States

**Keywords:** IDSS, SWAT, APEX, FARMSIM, Small-scale water management interventions, Environmental sustainability

## Abstract

This study investigates multi-dimensional impacts of adopting new technology in agriculture at the farm/village and watershed scale in sub-Saharan Africa using the Integrated Decision Support System (IDSS). Application of IDSS as an integrated modeling tool helps solve complex issues in agricultural systems by simultaneously assessing production, environmental, economic, and nutritional consequences of adopting agricultural technologies for sustainable increases in food production and use of scarce natural resources. The IDSS approach was applied to the Amhara region of Ethiopia, where the scarcity of resources and agro-environmental consequences are critical to agricultural productivity of small farm, to analyze the impacts of alternative agricultural technology interventions. Results show significant improvements in family income and nutrition, achieved through the adoption of irrigation technologies, proper use of fertilizer, and improved seed varieties while preserving environmental indicators in terms of soil erosion and sediment loadings. These pilot studies demonstrate the usefulness of the IDSS approach as a tool that can be used to predict and evaluate the economic and environmental consequences of adopting new agricultural technologies that aim to improve the livelihoods of subsistence farmers.

## Highlights

• We propose a modeling framework that assesses environmental and economical consequences of agricultural intensification.
• Agricultural interventions were evaluated using IDSS in two study sites in the Amhara region of Ethiopia.
• IDSS analyses indicate that a significant improvement in family incomes and nutrition can be achieved through the adoption of farming technologies such as irrigation technologies and nutrient management.

## 1 Introduction

The number of people who live below the poverty line in the world is estimated to have declined; however, there are still about 800 million people who are suffering from chronic hunger (FAO, 2013). Sub-Saharan Africa remains the region with the highest prevalence of undernourishment (FAO, 2013). The poor in sub-Saharan Africa usually live in rural areas (DFID, 2004, IFAD, 2011) and more than 90% earn less than $2/day (IFAD, 2011).

Agriculture is the leading economic sector in many developing countries despite production based on subsistence farming that suffers from low yields and high vulnerability due to climate change (Awokuse and Xie, 2015, Tibesigwa and Visser, 2015). In sub-Saharan Africa, 40–70% of rural households earn more than three-quarters of their income from on-farm sources (IFAD, 2011). For example, agriculture in Ethiopia accounts for 47% of GDP, 90% of exports and 85% of employment (IFAD, 2009). This suggests that investment in agriculture can contribute to food security and poverty reduction for the majority of the rural poor (DFID, 2004, WorldBank, 2008). Research across the world has proven that investment in agriculture can result in a sharp increase in economic development and poverty reduction (Gallup et al., 1998, Pretty et al., 2011, Thirtle et al., 2001, WorldBank, 2008). Green Revolution in Asia during the 1970s is one well-known example, where the adoption of science-based technology pulled Asia from the age of famine to regional food surplus within 25 years (Djurfeldt et al., 2005, Hazell, 2009).

Agriculture in sub-Saharan Africa is largely rainfed. Rainfed agriculture is the dominant source of staple food production (Cooper et al., 2008, FAO, 2011, Rosegrant et al., 2002) and covers 93% of the region’s agricultural area (CA, 2007, FAO, 2002). There is a large yield gap (i.e. the difference between what is actually harvested from farmers’ fields and what could potentially be achieved) in sub-Saharan agriculture. In tropical regions with reliable rainfall and sufficient nutrient application, average rainfed commercial agricultural yield exceeds 5–6 t/ha (CA, 2007, Rockström and Falkenmark, 2000) while the average rainfed yield in sub-Saharan Africa is less than 1.5 t/ha (Rosegrant et al., 2002).

Research has shown that the yield gaps in sub-Saharan Africa cannot be explained only by biophysical conditions (e.g. lower amounts of rainfall), but also due to sub-optimal management conditions (Licker et al., 2010, Rockström et al., 2002). Amede (2012) indicated that low yield in the rainfed agro-ecological landscapes of Ethiopia is typically not due to the lack of water alone rather a result of the inefficient management of water, soils, and crops. Supplementary irrigation combined with fertilizer application in a field experiment in the northern Burkina Faso increased the yield level three fold compared to the normal farmers’ practices (Fox and Rockstrom, 2003). In semi-arid Kenya, Barron and Okwach, (2005) showed that supplementary irrigation with improved nutrient application can increase average yield by 70%. In-situ water harvesting practices contribute to dry spell mitigation and yield improvements. Tied-ridges, also known as furrow damming, are found to improve barley yields by 44% compared to traditional tillage methods in the semi-arid region of northern Ethiopia (Araya and Stroosnijder, 2010). Moreover, soil and water management interventions improve land productivity by restoring degraded land (Lal, 2001, Reij et al., 2009, Vohland and Barry, 2009), maintain biodiversity (Norfolk et al., 2012, Pandey, 2001, Vohland and Barry, 2009), and limit nutrient loss from fields by controlling soil erosion (Gebreegziabher et al., 2009, McHugh et al., 2007).

There is growing interests in developing policies by governments and international development agencies on the implementation of small-scale soil and water management interventions in most of the sub-Saharan African countries (Gebregziabher et al., 2013). However, the scale of adoption of these technologies is very limited although there is proven evidence that small-scale interventions can improve agricultural production. The adoption of small-scale soil and water management interventions is affected by various socio-economic factors. Gebregziabher et al. (2013) reported that there exist specific main determinants that affect the adoption of rainwater management technologies in the Upper Blue Nile basin in Ethiopia such as household demographic characteristics (such as age and gender), participation in off-farm activities, migration, ownership of livestock, ownership of land, and other factors. Boyd et al. (2000) show how improved access to markets and increased producer prices stimulate investment in in-situ water harvesting systems at the household level in Tanzania. Munamati and Nyagumbo (2010) showed that resource status and gender determined the success of in-situ water harvesting in the Gwanda district of Zimbabwe.

**Fig. 1:**
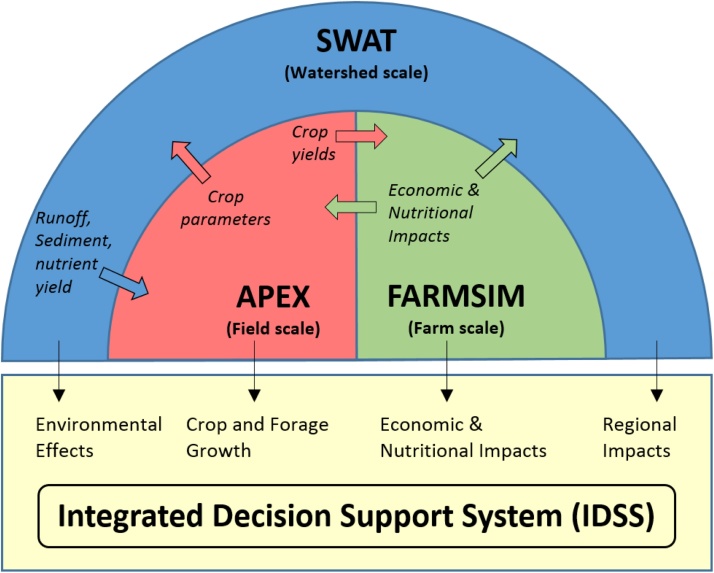
Schematic of the IDSS framework.

IDSS is comprised of a suite of previously validated, spatially explicit models (Soil and Water Assessment Tool (SWAT), Agricultural Policy/Environmental eXtender Model (APEX), and Farming Simulator (FARMSIM)), and databases that have been extensively applied in both U.S. and international settings. It provides an integrated approach linking production (APEX), economic (FARMSIM), and environmental (SWAT) consequences of the introduction of new technology, policy, and training for decision makers in agriculture at multiple temporal and spatial scales (Fig. 1). IDSS involves cropping systems analysis at the field scale, environmental risk assessment at the watershed scale, and nutritional and economic analyses of proposed interventions at the farm scale. The multi-scale and multi-aspects assessment is facilitated through passing information between SWAT, APEX, and FARMSIM such that proposed interventions are evaluated from different perspectives within a connected modeling framework. On the biophysical modeling side, SWAT and APEX share field/watershed information such as soil properties, historical weather data, or crop data. In addition, distributed parameters such as those related to water balance that are calibrated at the watershed scale are transferred to APEX for field scale analyses where observation data is unavailable. On the other hand, refined crop parameters at the field scale are transferred to SWAT to improve the calculation of evapotranspiration. Crop yield outputs on various intervention scenarios predicted by APEX are used in FARMSIM for feasibility assessment on the nutritional and economic point of view. Therefore, IDSS informs decision makers with unbiased assessment results.

SWAT was developed to predict the impact of land-management practices on water, sediment, and agricultural chemical yields in watersheds with varying soil, land use, and management conditions (Neitsch et al., 2012). SWAT can simulate hydrological cycles, vegetation growth, and nutrient cycling with a daily time-step by disaggregating a river basin into sub-basins and hydrologic response units (HRUs). HRUs are lumped land areas within the sub-basin comprised of unique land cover, soil and management combinations. SWAT has been applied in Ethiopian watersheds and demonstrated satisfactory performance (Dile et al., 2013, Dile and Srinivasan, 2014, Betrie et al., 2011, Setegn et al., 2010a, Setegn et al., 2010b, White et al., 2011). APEX is a biophysical simulation model that shares many of the attributes of SWAT. It is used to evaluate detailed crop management technologies and decisions that can affect agricultural production and environmental sustainability (soil, water, greenhouse gases, etc.) at the scales of individual fields, whole farms, or small watersheds (Ford et al., 2015, Gassman et al., 2010, Tuppad et al., 2009, Wang et al., 2012). FARMSIM is a farm level agro-economic model that simulates the nutritional uptake of farm family in a stochastic environment so the impacts of technology on the nutritional status of the farm family can be evaluated. FARMSIM is an extension of the Farm Level and Income Policy Simulation (FLIPSIM) model available in Microsoft Excel format which has been used extensively to simulate the impacts of alternative policies and farming systems on representative farms (e.g. Nyangito et al., 1995, Nyangito et al., 1996a, Nyangito et al., 1996b, Richardson and Clair, 1981, Richardson et al., 1983, Richardson et al., 2000). FARMSIM is explicitly programmed to simulate small holder farms in developing countries. In addition, it simulates the family unit as the “first” consumer of farm production.

Currently there are many biophysical and socio-economic models that show the impacts of small-scale interventions (e.g. Abildtrup et al., 2006, Chilonda and Van Huylenbroeck, 2001, Garg et al., 2012, van de Giesen et al., 2005). Moreover, there has been a number of models published for integrated hydrologic-agronomic-economic analysis (e.g. Cai et al., 2003, Lefkoff and Gorelick, 1990, Volk et al., 2008) but few model is capable of simulating hydrologic and agronomic components using comprehensive physically-based algorithms due to the complexity of the modeling system. The Spatial Decision Support System (Volk et al., 2008) is similar to IDSS in terms of implementing detail biophysical processes by integrating existing models and may be more advanced in assimilating data between process models. However, it is designed for ecologic-economic evaluation of land management or landuse changes. Little research has been done to show the impacts of these agricultural interventions using process-based simulation tools within an integrated decision framework on agricultural productivity and nutritional and economic impacts at the field scale and environmental consequences at the watershed scale. In this paper, the IDSS tool is applied to assess the effects of fertilizer, irrigation, genetically improved crop and crop rotation on production, environment, nutrition and economic wellbeing of small holder farmers in Fogera woreda of the Upper Blue Nile Basin, Ethiopia. This study demonstrates the use of IDSS for comprehensive assessment of complex agricultural technologies designed to increase food production, improve nutrition, enhance economic well-being, and minimize negative environmental consequences for small-holder farms.

## 2 Materials and methods

A pilot study was conducted for Ethiopia, in which the impacts of several levels of farming system intensification were evaluated at the kebele (i.e. the smallest administrative unit of Ethiopia) scale for productivity and nutritional/economic analyses and at the watershed scale for environmental impacts. The impacts of small-scale interventions were assessed in up to 13 scenarios including the current condition, fertilizer and irrigation management, cultivating generically improved varieties, and dry & wet season rotations combined with selected crops.

### 2.1 Description of study sites

**Fig. 2:**
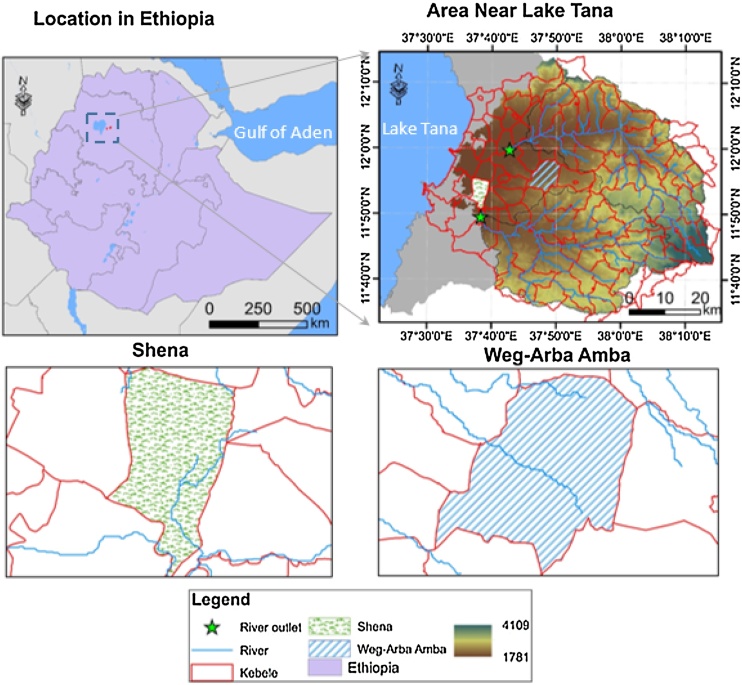
Location of study sites on the eastern shore of Lake Tana in Ethiopia.

Fogera woreda, located within the Gumera and Ribb river basins on the eastern side of Lake Tana in the South Gondar, Amhara region in Ethiopia (Fig. 2) was selected to demonstrate the capabilities of IDSS. A woreda is a second level administration unit in Ethiopia. The total land area of Fogerda woreda is 117,405 ha, with flat lands accounting for 76%, mountain and hills 11%, and valley bottoms 13%. (Gebey et al., 2012). Its total population is 233,529, with a rural population of 206,717. The male and female populations are similar in both rural and urban areas. The number of agricultural households is 42,746. The average land holding is about 1.4 ha, with minimum and maximum holdings of 0.5 and 3.0 ha respectively (ILRI, 2005; ILRI-IPMS Atlas, 2007). Two kebeles, Shena and Weg-Arba Amba, located within Fogera woreda were selected for case studies based on the following metrics.

• Close proximity to an all-weather road for access to markets
• Close proximity to a river or streams for possible irrigation
• Diverse existing land uses, including crop land, grazing land, and woodland
• One peasant associations (or kebeles) in the near-level Lake Tana plain and another in hilly region of the woreda

The Shena kebele is located on nearly level land within the Ribb river basin near Lake Tana at an elevation of 1774 m to 1850 m. Land uses include cultivated cropland, dense shrub land, open forest, grassland, exposed sand/soil, and seasonal swamp. Cropping systems are dominated by rice (*Oryza sativa* L.), finger millet (*Panicum miliaceum* L.), horticultural crops, and noug (*Guizotia abyssinica (L.f) Cass*); however, in recent years noug and finger millet are being replaced by maize and rice. Livestock include cattle, sheep and chickens. Weg-Arba Amba kebele is located in the Gumera river basin on rolling land with elevations ranging from 1774 m to 2153 m. The kebele is predominantly grassland with significant amounts of cropland, dense shrub land, and open forest. Cropping and livestock systems below 2000 m are dominated by maize, teff, finger millet, noug, vegetables, cattle, and goats. Above 2000 m barley, horse beans, potatoes, sheep and cattle are produced. Most of the crop land is located at higher elevations. Croplands are developed on slopes ranging from 6 to 23%; the two dominant crops in the area are corn and teff. Most farmers currently grow only one crop per year during the rainy season. In this area, crop residues are immediately removed for animal feed after grain harvest, reflecting current and anticipated future needs for animal fodder.

### 2.2 Biophysical modeling

Cropping system models of IDSS were implemented to simulate current low-input and possible future higher input cropping systems. In particular, SWAT was used to simulate hydrology, soil erosion, and Baseline crop yields for the Ribb and Gumera river basins and APEX was used to simulate several current and potential cropping systems for the Shena and Weg-Arba Amba kebeles for 32 years of weather, thus incorporating weather risk for alternative cropping systems. Irrigation and fertilizer inputs, and the environmental consequences of both current and high-input systems were also investigated using IDSS. The topography and land use/land cover data were derived from the United States Geological Survey (USGS) sources. Daily weather data were obtained from the NOAA-USGS Climate Forecast System Reanalysis (CFSR) global daily weather database. This 32-year (1979–2010) daily weather data contains precipitation, minimum and maximum temperatures, solar radiation, wind speed, and humidity (available at: globalweather.tamu.edu, last accessed on December 9, 2015). Soils data were obtained from the FAO 1:1 million world soils map and associated soil pedon data base (MoWR, 2009). Calibrated SWAT parameters were transferred to APEX to better simulate water balance at the field scale. Water quality data was not available and therefore the model was not refined for water quality related parameters. In turn, APEX crop parameters were transferred to SWAT to synchronize the two models on predicting water balance.

To demonstrate the role of the IDSS models on analyzing the impacts of crop management on crop growth and soil erosion in different landscapes, two levels of crop management were simulated for all the subbasins of the Rib and Gumera watersheds. The two input levels were: (1) the Baseline (low-input) maize, teff, and maize-rice-onion cropping system in use in the region, and (2) the same cropping systems with additional nitrogen fertilizer and irrigation in the dry season (with rates determined by the automatic fertilizer and irrigation routines in SWAT).

For simulating alternative cropping systems using APEX, a deep alluvial soil was used for the Shena kebele site and a shallow rocky soil was used for the Weg-Arba Amba kebele. The maize and teff fields on steep slopes were simulated with shallow clean tillage, no structural conservation practices, and complete residue removal (for animal feed) based on local practices. Rock barriers at the edges of many of the region’s small fields produce field-edge sedimentation and reduce sediment delivery to streams in the region, which was represented in APEX by reduced slope lengths. To assess fertilizer and irrigation requirements and their effects in traditional grain cropping systems, continuous maize and continuous teff production were modeled as summarized in Table 1.

### 2.3 Economic and nutrition analysis

A farm scale Monte Carlo simulation model, FARMSIM, was used to perform economic and nutrition analysis for the two study regions. Two representative farms were simulated for the base and two alternative technology farming systems or scenarios. The FARMSIM model simulates a representative farm for five years (2013–2017) using stochastic market prices, crop yields, and livestock production values. The model is recursive in that the financial values at the end of 2013 are the beginning financial values for the start of 2014. Cattle, sheep, goats, and chickens are included in the model and their numbers are simulated over time based on parameters for fractions of animals consumed, died, culled, and stochastic birthrates.

Data to simulate farms in the two kebeles were provided by local experts in the Amhara Region and International Water Management Institute (IWMI) based in Addis Ababa, Ethiopia. The aggregate data for the kebele were used to describe and simulate each kebele as a representative farm due to problems associated with simulating individual small-holder farms such as heterogeneity and possibilities of outliers. The Shena farm represents the 2071 ha of cropland and 2151 families for the kebele. The Weg-Arba Amba farm represents 1514 families and 3640 ha of cropland (Table 2).

The APEX model was simulated for 32 years using local weather data for 1979–2010 for each farming system to estimate annual crop yields under alternative weather conditions. The resulting 32 simulated grain and forage yields provided necessary data for FARMSIM to estimate probability distributions (PDFs) for yield since crop yield is a stochastic variable in FARMSIM. Stochastic annual yields for crops and forage in FARMSIM was simulated as multivariate empirical (MVE) PDFs maintaining the observed correlation for the stochastic yields generated by APEX (Richardson et al., 2000). Three MVE PDF’s were developed for each farm as different yield distributions were used for their respective scenarios/farming system.

Results of the FARMSIM model are presented in different statistical variables, graphs and stoplight chart. The statistical variables include mean, standard deviation, coefficient of variation, minimum and maximum. The graphical presentations include probability density functions and cumulative probability density functions. The stoplight chart is simply way of presenting probabilities of getting different socio-economic outcomes using color coded charts. The color codes include green, yellow and red. Green represents the probability of getting the best outcome; yellow is the probability of getting moderate outcome and red is probability of getting the least socio-economic outcome.

### 2.4 Assumptions and limitation

Databases used in the analysis were taken from available sources and expert opinions. A variety of modeling related issues such as roads and markets and other infrastructure, training and education, and enabling policies that might be required to bring the proposed interventions to practice were not considered in the current study. The literature on technology indicates that the rate of adoption varies widely for alternative technologies. For the current analysis it was assumed that the farming systems interventions are adopted at the rate of 25% and this adoption rate is constant throughout the planning horizon. The model is capable of simulating any assumed adoption curve where the rate of adoption changes each year, such as, 5%, 10%, 15%, 20% and 25% over the 5 year planning horizon. To test the sensitivity of the adoption rate assumption the Baseline + N farming system was also simulated using a 50% adoption rate.

## 3 Results and discussion

### 3.1 Watershed scale simulations

For the Gumera and Rib river watersheds, mean stream flows totaled almost 60% of the mean total precipitation as the result of large amounts of runoff and subsurface flow during the wet season. These results are consistent with past studies in the Blue Nile basin (Bitew and Gebremichael, 2011, Setegn et al., 2011, Tibebe and Bewket, 2011) and suggest that, depending on location within the watersheds, ample water may be available, either for capture in small reservoirs or in shallow aquifers, for use as supplemental irrigation during the dry season. In fact implementation of small reservoirs depends on the suitability of biophysical factors such as land use, soil, slope etc. (e.g. Dile et al., 2016). This study assesses the potential water resources to be captured by small reservoirs, but didn’t identify locations that are suitable for implementing small reservoirs. Moreover, implementation of small reservoirs may also have impacts on the downstream social and ecological systems. Therefore, assessing suitable areas for implementation of small reservoirs and their impact on the downstream social and ecological system warrants further investigation.

**Fig. 3:**
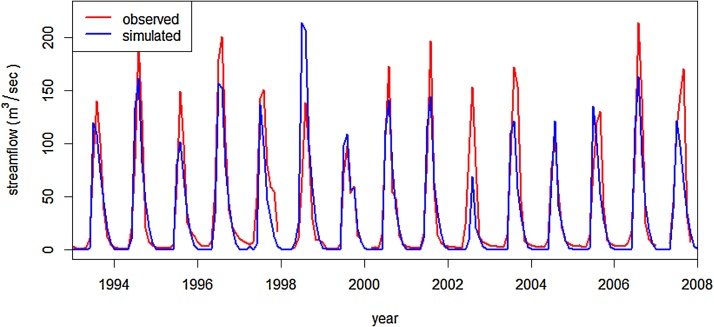
Monthly measured and simulated stream flows in Gumera River observed near its outlet to Lake Tana.

The accuracy of multi-year estimation of monthly stream flows was evaluated by comparing SWAT outputs with measured data by the Ministry of Water, Irrigation & Electricity of Ethiopia at the Gumera river gauging station. The SWAT model parameters are calibrated using observed stream flow at the Gumera river gauging station, which is the closest gauging station to the study watershed. Daggupati et al. (2015) recommended calibrating ungauged watersheds using observed stream flow from a close-by gauged watershed. In many cases systematic errors were identified and addressed using the SUFI method, a sophisticated optimization approaches available in SWAT-CUP (Abbaspour, 2015). Fig. 3 shows measured and simulated monthly flows in Gumera River near its outlet to Lake Tana. The results (R^2^ = 0.76 and NSE = 0.75) indicate that using globally available weather and landscape data, SWAT reasonably simulated monthly stream flows in this region of Ethiopia.

**Fig. 4:**
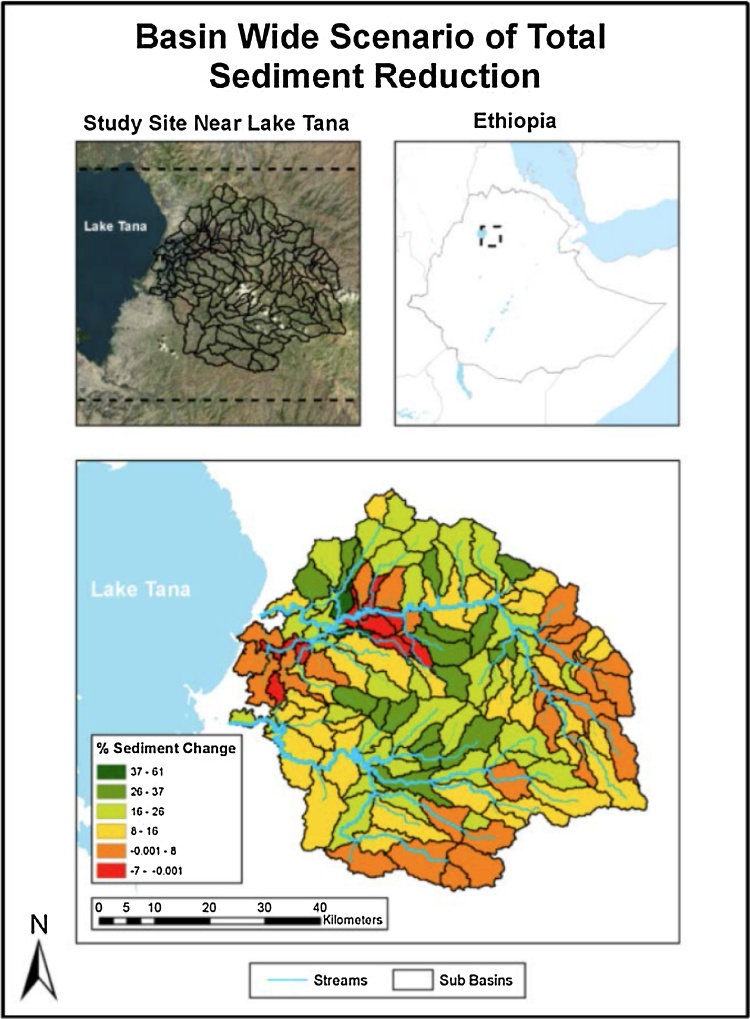
Impacts of changing from low to high N fertilizer rates for crop rotations including maize, teff, rice, and onion for the same crop rotations on sediment delivery. Darker green colors indicate greater reductions in sediment yield. (For interpretation of the references to colour in this figure legend, the reader is referred to the web version of this article.)

SWAT results averaged over the entire Rib and Gumera river basins are summarized in Table 3. As reported by Ethiopian agricultural experts and other hydrologic studies of the region (Betrie et al., 2011, Setegn et al., 2009, Setegn et al., 2010a) significant erosion rates were estimated. The impacts of crop management scenarios on soil erosion are shown in Fig. 4; green indicates larger reductions in sediment yield while red indicates small or no reductions in sediment yield.

As shown in Fig. 4, a substantial increase in nitrogen fertilizer application results in a notable reduction in sediment delivery to streams in the sub-basins of the Rib and Gumera watersheds. Reduced erosion resulted from large increases in crop leaf area and biomass, which would reduce raindrop energy at the soil surface. Soil erosion happens because of the erosive forces of rain drops and surface flow of water (Arnold et al., 2012). Raindrop impact can detach soil particles on unprotected land surfaces between rills and initiate transport of these particles to the rills (Arnold et al., 2012). As the leaf area increases, the canopy cover increases and thereby reduces the erosive force of the raindrop. As such an increase in leaf area index and biomass, reduces soil erosion. These effects were greater on the sloping croplands between the Gumera and Rib watersheds (the green areas in the middle of the figure) than on the more level lands near the outlets of the watersheds or in the sub-basins with lesser amounts of cropland in the eastern and southern parts of watershed. In addition, the result indicates that sediment delivery to Lake Tana would be substantially reduced by implementation of more intensive cropping systems with appropriate conservation practices.

### 3.2 Kebele scale simulations

#### 3.2.1 Weg-Arba amba kebele

The Weg-Arba Amba kebele occupies an upland site with slopes ranging from 6 to 23%; the two dominant crops in the area are corn and teff. Most farmers currently grow only one crop per year during the rainy season. In this area, crop residues are immediately removed for animal feed after grain harvest, reflecting current and anticipated future needs for animal fodder. The results of these APEX simulations are summarized in Table 4. The Baseline scenario represented typical low-input continuous maize and continuous teff cropping systems for the Weg-Arba Amba kebele. As might be expected in a low-input farming system of this type, grain yields were quite low and strongly limited by nitrogen fertility. This is reflected by the large values of metrics for simulated nitrogen stress (NS) and water stress (WS).

IDSS/APEX crop model is sensitive to the abiotic stresses such as NS and WS that usually limit crop yields, and is able to dynamically estimate these stresses and simulate application of fertilizers and irrigation to minimize them. For example, for the Baseline + N + I scenario, IDSS applied 202 kg/ha of N fertilizer and 110 mm of supplemental irrigation for Maize (Table 4). This caused mean grain yields to increase from 2.4 to 8.8 tons/ha and greatly reduced the metrics for nitrogen (NS) and water stress (WS) substantially. Similar results were found for teff. Another effect of the N + I scenario was a small reduction in soil erosion due to the greater leaf area index of the well-fertilized crop. For the N + I + V scenario, the estimated crop yields increased with essentially no impact on stresses as the scenario represents genetically improved crops.

The Maize-Chickpea Relay Crop scenario caused a slight reduction in maize yield due to inter-crop competition. In addition, since all chickpea residue was removed after harvest for animal fodder, no effect of residual legume nitrogen was observed on maize yields. Despite the slight negative effect on maize yields, adding an irrigated, dry-season chickpea crop to the Baseline maize cropping system increased protein production with no additional fertilizer inputs. The Maize-Squash Relay Crop was extension to the Baseline unirrigated maize cropping system by adding squash to increase fresh vegetable production. Because the maize crop had taken up virtually all the available soil nitrogen by the time the squash was planted, and the rainy season normally ended before the squash matured, additional fertilizer nitrogen and irrigation were needed to obtain adequate squash yields.

As the shallow and rocky soils of the Weg-Arba Amba kebele suggest, severe soil erosion is occurring in the steep lands of the Ethiopian highlands due to little structural erosion control and over-grazing of crop residue (Betrie et al., 2011, Setegn et al., 2010a, Setegn et al., 2009). These simulations assumed clean tillage, no structural erosion controls, and removal of all crop residues for animal feed estimated annual erosion rates of over 20 tons per hectare. Even with adequate fertilizer, runoff (Q) plus subsurface return flow to streams are large, approaching total crop evapotranspiration (ET). This demonstrates that plenty of water is available for capture in small reservoirs for irrigation during the dry season. Simple low-pressure drip irrigation systems may be a useful technology to move farm families toward small-scale but intensive production of additional vegetable and/or grain legume crops.

#### 3.2.2 Shena kebele

The Shena kebele site is located on the near-level floodplain near the shores of Lake Tana. In addition to simulating the low-input Baseline maize-rice-onion rotation, the effects of increasing levels of nitrogen fertilizer and growing improved crop varieties were assessed. A fourth two-year cropping system, consisting of a two-year maize-rice-onion-soybean-rice-onion rotation, was simulated to examine the effects of including a grain legume to increase protein production without increasing nitrogen fertilizer rates. Results of these simulations are summarized in Table 5.

The Maize-Rice-Onion Baseline scenario was a three-crop rotation of maize, rice, and onion. The rice was grown in paddies during the rainy season and received ample rainfall to maintain saturated or near-saturated soil conditions. Maize and onion were grown in the dry season and were irrigated at rates (IRGA) determined dynamically by APEX. Fertilizer rates for the Baseline cropping system were typical for the region, but APEX found that for this cropping system grain and onion yields are greatly limited by nitrogen stress (NS). In the Baseline + N scenario, fertilizer N requirements and crop response were investigated. In response to crop N stress, APEX increased average annual fertilizer N application from the 101 kg/ha Baseline rate to 387 kg/ha. The additional fertilizer reduced N stress (NS) to near zero and increased average yields of maize, rice, and onion by 246%, 143% and 144%, respectively, and almost doubled average annual protein production by the maize and rice crops. In addition, crop residue yields increased substantially, providing additional fodder for livestock.

The effects of growing crops with 15% greater harvest indexes were also simulated with increased fertilizer N (Baseline + N + V scenario) to examine the sensitivity of varietal improvement. As expected, APEX simulated greater yields, greater protein production, and slightly higher average nitrogen fertilizer requirements (402 kg N/ha) when varieties with increased yield potential were used (Table 5).

There was a substantial increase in protein production when maize was replaced with soybean every other year in the Baseline cropping system (without additional fertilizer nitrogen). Introduction of soybean slightly increased maize and rice yields compared to the Baseline maize-rice-onion rotation, but average annual protein production of the entire cropping system increased substantially (1628 kg/ha). This highlights the importance of including grain legumes in crop rotations to increase protein production of cropping systems where fertilizer nitrogen is limited.

### 3.3 Economic and nutritional impacts

#### 3.3.1 Weg-Arba Amba kebele

The changes in economic benefits of the Weg-Arba Amba kebele for alternative farming systems are reported in Table 6. The Weg-Arba Amba kebele is non-irrigated in the Baseline and Alternative scenarios and has a 7650 Birr average annual net cash farm income (NCFI) per family which is equivalent to $352 USD based on the exchange rate in June 2016. For the Baseline + N +I scenario NCFI averages 103,080 Birr and for the Baseline + N + I + V scenario the value is 114,210 Birr. The increases in NCFI are due to higher grain and associated forage yields. Increased forage yields and reduced yield risk lead to increased livestock production and receipts. Average ending cash reserves in 2017 are projected to increase from 12,390 Birr in the Baseline to 552,260 Birr for the Baseline + N + I + V scenario.

The nutritional benefits from improved yields and higher average annual NCFI per family are observed in terms of increased average daily consumption of energy, protein, fat, calcium, iron and vitamin A. The Baseline + N + I + V scenario leads to a 9.5% increase in average daily consumption of energy (calories) for a family in the Weg-Arba Amba kebele. FARMSIM uses the Organization for Economic Co-operation and Development (OECD) definition of an adult equivalent (AE) which represents a weighted aggregation of a family unit’s consumption. An AE counts the first adult as 1 and each additional adult at 0.7 and counts children under 15 at 0.5.

**Fig. 5:**
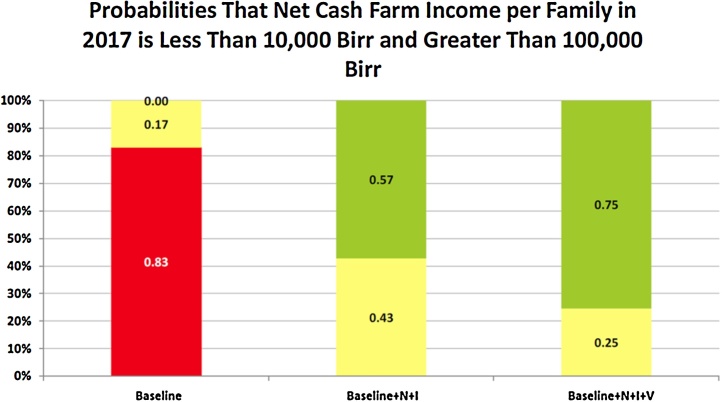
StopLight Chart for per Family NCFI on a Weg-Arba Amba Farm for Alternative Farming Systems. The StopLight function presents the probability of: (a) exceeding the upper target (green); (b) being less than the lower target (red), and (c) observing the values between the targets (yellow). (For interpretation of the references to colour in this figure legend, the reader is referred to the web version of this article.)

The stochastic capabilities of FARMSIM provide probabilistic results for the output variables. The alternative farming systems shift the NCFI probability distribution to the right which reduces the chance of low average annual NCFI per family. The StopLight chart in Fig. 5 shows that there is an 83% chance annual NCFI is less than 10,000 Birr for the Baseline case. The StopLight Chart compares the target probabilities for one or more risky alternative (or scenarios) where the user must specify the two targets (Lower and Upper target). For the Baseline + N + I scenario the probability of annual NCFI being below 10,000 Birr is zero and there is a 57% chance that annual NCFI is greater than 100,000 Birr. Under a high technology adoption scenario (Baseline + N + I + V), the probability of annual NCFI exceeding 100,000 Birr is 75%.

**Fig. 6:**
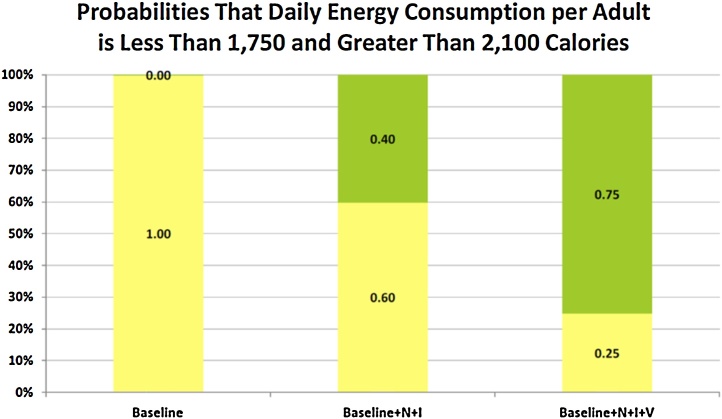
StopLight Chart for Daily Energy Consumption per Adult Equivalent on a Weg-Arba Amba Farm for Alternative Farming Systems.

The StopLight chart for daily energy consumption per AE is presented in Fig. 6. Introduction of irrigation and proper fertilization (Baseline + N + I) decreases the probability that daily energy consumption is less than 2,100 cal per AE from 100% to 60%. The high technology scenario (Baseline + N + I + V) further improves the probability of adequate energy consumption by increasing the probability of daily caloric intake per AE greater than 2,100 cal to 75 percent. Similarly, the daily consumption of protein, fat, calcium, iron and vitamin A increases as yields improve with the alternative farming systems.

#### 3.3.2 Shena kebele

The average annual NCFI per family under the Baseline is estimated at 19,010 Birr for the Shena kebele (Table 7). By optimizing crop production under the Baseline + N + I scenario, the average annual NCFI is 51,570 Birr and under the high technology adoption scenario (Baseline + N + I + V) the average annual NCFI is 58,960 Birr. The increases in NCFI are due to higher grain and onion yields and forage yields. Increased forage yields and reduced yield risk lead to increased livestock production and receipts. The increased average annual NCFI per family would lead to increases in ending cash reserves per family in 2017 (67,700 Birr for Baseline and 204,800 Birr for Baseline + N + I).

**Fig. 7:**
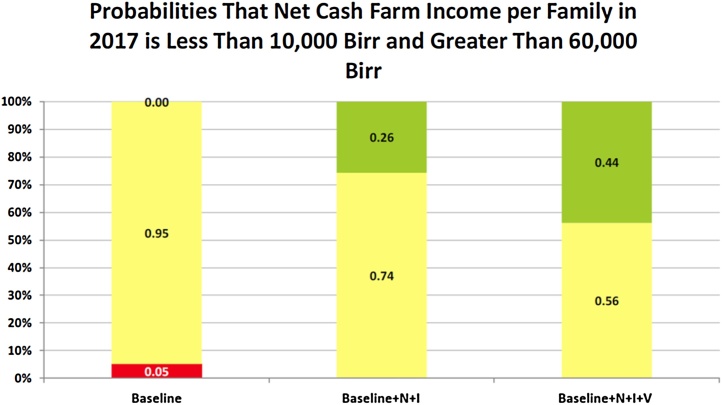
StopLight Chart for per Family NCFI on a Shena Farm for Alternative Farming Systems.

The average daily consumption of calories for an AE is estimated at 1,913 cal for the Baseline, 1990 for the Baseline + N + I and 2071 for the Baseline + N + I + V scenario (The OECD defines an AE as a weight for aggregating a family unit. It counts the first adult as 1 and each additional adult at 0.7 and counts children under 15 t 0.5). Similarly, the daily consumption of protein, fat, calcium, iron and vitamin A increases as yields improve with the alternative farming systems. The probabilities of annual NCFI being less than 10,000 Birr and greater than 60,000 Birr are summarized in a StopLight chart in Fig. 7. Under the Baseline there is a 5% chance that annual NCFI for a farm family is less than 10,000 Birr and a zero chance it exceeds 60,000 Birr. For the Baseline + N + I scenario the probability that annual NCFI for a farm family exceeds 60,000 Birr is 26% and for the Baseline + N + I + V scenario the probability is 44%. Increased technology reduces the probability of low NCFI to zero and increases the probability of high NCFI.

**Fig. 8:**
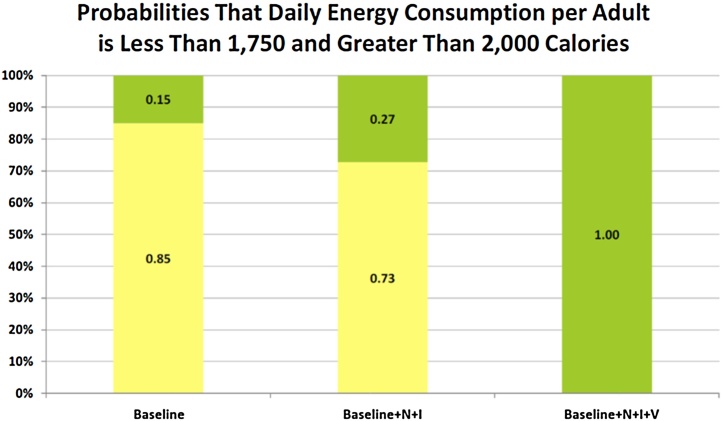
StopLight Chart for Daily Energy Consumption per Adult Equivalent on a Shena Farm for Alternative Farming Systems.

The StopLight chart for daily energy consumption per AE is presented in Fig. 8. The introduction of improved production practices decreased the probability of energy deficiency in the diet. There is 85% chance that energy consumption per AE is between 1750 and 2000 cal per day for the Baseline, and a zero probability of energy consumption less than the high target value (2000 cal) for the Baseline + N + I + V scenario. Although stated as AE, the probable deficiency would likely have most serious consequences for the physical and cognitive development of children in the family. The Baseline + N + I scenario actually provides a 27% chance that average daily energy consumption per AE will exceed 2000 cal. The daily consumption of protein, fat, calcium, iron and vitamin A increases as yields improve with the alternative farming systems.

Introduction of proper plant fertilization and irrigation has the potential for greatly improving net income and nutritional intake for small-holder farmers in the Shena and Weg-Arba Amba kebele. Greater benefits will be observed for the Weg-Arba Amba farms because these farms are presently non-irrigated; average NCFI per family increases from 7650 to 103,080 Birr per year and net present value increases 400%. The Shena farms use irrigation there will still be a significant increase in average net income (27%) and net present value (180%) by optimizing their plant nutrition program. Improvements in farming systems lead to an increase in daily intake of nutrients for an AE. The nutritional improvements are smaller (5%–10%) than the income increases because the kebele are producing adequate levels of grain and families are consuming adequate levels of nutrients in the Baseline case. Additionally, during years of low yields adequate cash should be available in the Baseline case to purchase food to meet current desired levels of consumption. Prolonged droughts would pose a significant problem because cash reserves would be reduced to zero after the first few years of drought.

#### 3.3.3 Economic impacts for the Lake Tana Basin watershed

The economic impacts to the watershed analyzed with SWAT for the alternative farming systems are projected based on the per ha average annual net cash farm income projected for the representative farms and the number of ha of land with similar slope in the watershed. The watershed used for the analysis contains the Gumera and Ribb river basins. The watershed has about 8000 ha of cropland similar to less than 10% slope land in the Shena kebele. It is estimated that 750,000 ha of cropland that can be potentially irrigated in the watershed has from 10% to 20% slope like the maize/teff land in the Weg-Arba Amba kebele. Scaling up the average annual NCFI for the watershed, based on ha in the two kebeles, projected NCFI per hectare, and number of similar cropland ha is not ideal but it gives gross estimate of the economic impacts to the watershed.

The annual economic impacts, assuming widespread adoption of the Baseline + N + I farming system, is expected to be 11.2 million Birr and, for adoption of the Baseline + N + I + V farming system, is expected to be 13.3 million Birr (Table 8). The increases in average NCFI are largely due to the significant increase in net income for the non-irrigated maize/teff rotation and the very large number of ha planted to this rotation in the watershed. If full adoption in a short period was observed, there would most likely be a negative price impact which would decrease these estimated increases in NCFI. However, given a more normal adoption process the price effects will be much smaller as markets and population adjust to improved quantity and quality of food.

## 4 Conclusion

An integrated assessment was conducted using IDSS for multi-scale analyses of the impact of introducing new technology at the farm/village and watershed levels of scale. Regions that are agriculturally important in Ethiopia were selected and analyzed from watershed down to the representative farm in two kebeles. SWAT, APEX, and FARMSIM were integrated by exchanging the output of one model to the other model as input for comprehensive assessment of agricultural technologies that are designed to increase food production, improve nutrition, enhance economic well-being, and minimize negative environmental consequences for small holder farms.

Even with conservative estimates of adoption, the results of the interventions were positive. The interventions implemented in this study are optimized to enhance the sustainable production of food under the conditions of the two locations and their related watersheds. The selection of the two sites was made to assure a reasonable access to markets and to compare different opportunities for the introduction of irrigation methods. Clearly, the successful introduction of the new farming practices and technologies would require substantial development of infrastructure such as roads and marketing support, supportive policy environments, and active programs such as those now being undertaken in Ethiopia to train and motivate changes in social mores for adoption of new technologies. This study includes the impact of traditionally low adoption rates, which might be enhanced by a more informed population.

The case studies demonstrate that the IDSS can be a useful tool to predict and evaluate the consequences of various interventions to improve the livelihoods of subsistence farmers while evaluating the environmental consequences at multiple levels of scale. The results demonstrate the ability to predict the outcomes of interventions using quantitative stochastic methods. IDSS provides an integrated systems approach to assessment of new investment options, including analysis of water use and protection to feed future populations. The ability to concurrently assess production, environmental, and economic and nutritional consequences of options for sustainable increases in production of food and use of scarce natural resources using an integrated modeling approach adds unique value to the current knowledge in agricultural research.

## Figures and Tables

**Table 1:** Scenarios of agricultural interventions simulated with APEX.

Scenarios	Description	Sites
Baseline	current low-input, rainfed cropping system and management practices	Both
Baseline + N + I	a more intensive cropping system in which APEX was allowed to estimate and apply fertilizer and irrigation necessary to reduce nitrogen and water stress to near-zero	Both
Baseline + N + I + V	genetically improved varieties represented by crops with a 15% increase in harvest index	Both
maize-chickpea	maize-chickpea relay cropping systems with current fertilizer inputs but supplemental irrigation of the chickpea during the dry season	Weg-Arba Amba
maize-squash	maize-squash relay cropping systems with current fertilizer inputs but supplemental irrigation of the squash during the dry season	Weg-Arba Amba
Maize-Rice-Onion-Soybean-Rice-Onion	2-year rotation of maize-rice-onion-soybean-rice-onion with current fertilizer inputs but supplemental irrigation of onion during the dry season	Shena

**Table 2:** Socio-economic characteristics for Shena and Weg-Arba Amba kebeles.

	Shena	Weg-Arba Amba
Number of families	2151	1514
Cropland (Ha)	2071^a^	3624

Crops:		
Maize (Ha)	1800	914
Rice (Ha)	2071	–
Onion (Ha)	1800	–
Teff (Ha)	–	2710

Livestock:		
Cows	4174	1893
Oxen	4611	1788
Hens	6000	3020
Ewes	700	150
Nannies	–	1853

a The total area of the cropland does not match the sum of the areas for individual crops because of continuous rotation of multiple crops.

**Table 3:** Simulated cropping systems, vegetation types, and mean annual hydrologic and soil erosion components for the combined Gumera and Rib watersheds. Continuous maize and teff crops are grown in the main wet season without irrigation.

Land Use	Area	Precipitation	Irrigation	Runoff	Subsurface runoff	ET	Erosion
	(km^2^)	(mm)	(mm)	(mm)	(mm)	(mm)	(t/ha)
Cropland							
Maize	1382	1303	0	402	351	525	85
Teff	944	1294	0	372	409	483	114
Maize-Rice-Onion	8	1313	639	386	237	1139	8
Grazing land	1049	1301	0	356	399	517	15
Forest land	5	1345	0	269	488	557	<1
Village	3	1278	0	472	279	503	<1

**Table 4:** Weg-Arba Amba kebele simulated cropping systems.

Treatment^a^	Yield	Residue	FN	FP	Q	WYld	ET	IRGA	Y	WS	NS
	(t/ha)	(t/ha)	(kg/ha)	(kg/ha)	(mm)	(mm)	(mm)	(mm)	(t/ha)	(d)	(d)
Continuous Maize											
Baseline	2.4	2.3	56	40	406	681	562	0	30	14	65
Baseline + N + I	8.8	8.0	202	40	389	647	702	110	28	6	2
Baseline + N + I + V	10.2	6.8	202	40	389	646	703	110	28	6	2

Continuous Teff											
Baseline	0.6	1.1	24	17	372	648	596	0	33	23	31
Baseline + N + I	4.4	7.3	152	17	376	656	726	150	28	7	0
Baseline + N + I + V	5.0	6.8	152	17	376	656	726	150	28	7	0

Maize-Chickpea Relay Crop											
Maize	2.2	2.0	56	40	334	659	635	0	23	16	66
Chickpea	1.0	1.1	0	0	–	–	–	66	–	14	2

Maize-Squash Relay Crop											
Maize	2.1	2.0	56	40	331	666	739	0	24	16	67
Squash	0.3	0.6	40	0	–	–	–	184	–	3	46

a Grain/fruit yields (Yield), residue yields (Residue), fertilizer nitrogen (FN), fertilizer phosphorus (FP), runoff (Q), runoff + return flow to stream (WYld), evapotranspiration (ET), irrigation (IRGA), soil erosion (Y), water stress (WS), and nitrogen stress (NS).

**Table 5:** Shena kebele simulated irrigated maize, rice, Soybean, and onion rotation.

Treatment^a^	Yield	Residue	FN	FP	Q	WYld	ET	IRGA	Y	WS	NS	Protein
	(t/ha)	(t/ha)	(kg/ha)	(kg/ha)	(mm)	(mm)	(mm)	(mm)	(t/ha)	(d)	(d)	(kg/ha)
Baseline												
Maize	3.7	3.4	23	0	266	363	1591	591	0.7	6	47	300
Rice	4.2	4.3	55	23	–	–	–	0	–	1	45	352
Onion	1.8	0.1	23	0	–	–	–	111	–	1	21	–

Baseline + N + I												
Maize	9.1	8.6	178	0	272	370	1597	605	0.7	7	1	737
Rice	6	6.2	138	23	–	–	–	0	–	1	1	510
Onion	2.6	0.2	71	0	–	–	–	108	–	1	0	–

Baseline + N + I + V												
Maize	10.5	7.3	179	0	272	370	1607	604	0.7	7	1	850
Rice	6.9	5.5	150	23	–	–	–	0	–	2	1	586
Onion	3.7	0.1	73	0	–	–	–	117	–	2	0	–

Irrigated Maize-Rice-Onion-Soybean-Rice-Onion Rotation (2 Years)												
Maize	3.9	3.6	23	0	266	363	1631	578	0.6	3	47	316
Soybean	3.7	8	0	0	–	–	–	684	–	5	0	1628
Rice	4.7	4.9	55	23	–	–	–	0	–	3	34	400
Onion	1.9	0.1	23	0	–	–	–	111	–	1	19	–

a Simulated 2-year rotation of maize-rice-onion-soybean-rice-onion. Grain/bulb yields (Yield), residue yields (Residue), fertilizer nitrogen (FN), fertilizer phosphorus (FP), runoff (Q), runoff + return flow to stream (WYld), evapotranspiration (ET), irrigation (IRGA), soil erosion (Y), water stress (WS), nitrogen stress (NS), grain protein content (Protein).

**Table 6:** Economic and nutrition impacts of alternative farming systems for the Weg-Arba Amba kebele.

	Baseline	Base + N + I	Base + N + I + V
	(*1000 Birr)	(*1000 Birr)	(*1000 Birr)
Average values per family			
Net Present Value	100.7	408.74	447.44
Average Net Cash Farm Income	7.65	103.08	114.21
Average Ending Cash Reserves	12.39	492.71	552.26
Energy per Adult (cal/day)	1884	2091	2133
Protein per Adult (grams/day)	55.6	60.1	61.1
Fat per Adult (grams/day)	35.7	40	40.4
Calcium per Adult (grams/day)	0.25612	0.29144	0.29711
Iron per Adult (grams/day)	0.01718	0.01878	0.0192
Vitamin A per Adult (grams/day)	0.00118	0.0012	0.0012

*Note*: the values in table are multiples of 1000.

**Table 7:** Economic and nutrition impacts of alternative farming systems for the Shena kebele.

	Baseline	Baseline + N + I	Base + N + I + V
	(1000 Birr)	(1000 Birr)	(1000 Birr)
Average values per family			
Net Present Value	135.14	243.79	272.46
Average Net Cash Farm Income	19.01	51.57	58.96
Average Ending Cash Reserves	67.39	204.80	247.45
Energy per Adult (cal/day)	1913	1990	2071
Protein per Adult (grams/day)	58.5	60.9	62.4
Fat per Adult (grams/day)	38.20	41.20	42.00
Calcium per Adult (grams/day)	0.32600	0.38401	0.38560
Iron per Adult (grams/day)	0.01731	0.01757	0.01807
Vitamin A per Adult (grams/day)	0.00161	0.00163	0.00163

*Note*: the values in table are multiples of 1000.

**Table 8:** Economic Benefits to the Watershed from Adoption of Alternative Farming Systems.

	Average annual Net Cash Farm Income			
Crop Rotation/Slope	Ha	Base	Base + N + I	Base + N + I + V
	(ha)	(1000 s Birr)		
Onion/Corn/Rice; fields < 10% slope	8000	76	207	237
Change from Base			131	160
Corn/Teff; fields 10–20% slope	750,000	890	11,995	13,290
Change from Base			11,105	13,214
Total Change in NCFI for Watershed			11,236	13,374
